# Novel Approaches for The Development of Live Attenuated Influenza Vaccines

**DOI:** 10.3390/v11020190

**Published:** 2019-02-22

**Authors:** Pilar Blanco-Lobo, Aitor Nogales, Laura Rodríguez, Luis Martínez-Sobrido

**Affiliations:** Department of Microbiology and Immunology, School of Medicine and Dentistry, University of Rochester, Rochester, New York, NY 14642, USA; piblanlo@gmail.com (P.B.-L.); aitor_nogales@hotmail.com (A.N.); laurita85oviedo@hotmail.com (L.R.)

**Keywords:** influenza virus, influenza vaccines, influenza inactivated vaccine (IIV), live-attenuated influenza vaccine (LAIV), influenza reverse genetics, recombinant influenza virus, immunogenicity, protection efficacy

## Abstract

Influenza virus still represents a considerable threat to global public health, despite the advances in the development and wide use of influenza vaccines. Vaccination with traditional inactivate influenza vaccines (IIV) or live-attenuated influenza vaccines (LAIV) remains the main strategy in the control of annual seasonal epidemics, but it does not offer protection against new influenza viruses with pandemic potential, those that have shifted. Moreover, the continual antigenic drift of seasonal circulating influenza viruses, causing an antigenic mismatch that requires yearly reformulation of seasonal influenza vaccines, seriously compromises vaccine efficacy. Therefore, the quick optimization of vaccine production for seasonal influenza and the development of new vaccine approaches for pandemic viruses is still a challenge for the prevention of influenza infections. Moreover, recent reports have questioned the effectiveness of the current LAIV because of limited protection, mainly against the influenza A virus (IAV) component of the vaccine. Although the reasons for the poor protection efficacy of the LAIV have not yet been elucidated, researchers are encouraged to develop new vaccination approaches that overcome the limitations that are associated with the current LAIV. The discovery and implementation of plasmid-based reverse genetics has been a key advance in the rapid generation of recombinant attenuated influenza viruses that can be used for the development of new and most effective LAIV. In this review, we provide an update regarding the progress that has been made during the last five years in the development of new LAIV and the innovative ways that are being explored as alternatives to the currently licensed LAIV. The safety, immunogenicity, and protection efficacy profile of these new LAIVs reveal their possible implementation in combating influenza infections. However, efforts by vaccine companies and government agencies will be needed for controlled testing and approving, respectively, these new vaccine methodologies for the control of influenza infections.

## 1. Introduction

### 1.1. Influenza A Virus (IAV)

**Figure 1:**
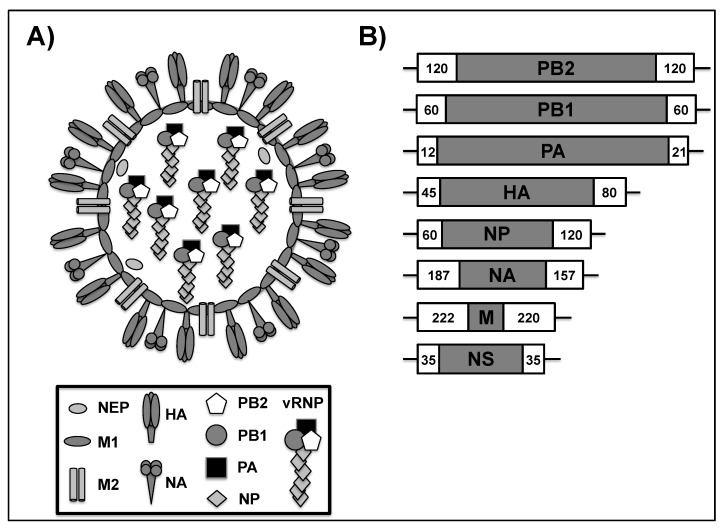
Influenza A virus (IAV) virion structure and genome organization. A) Virion structure: IAV is an eight-segmented, negative-sense, single-stranded RNA enveloped virus surrounded by a lipid bilayer that contains three viral glycoproteins: hemagglutinin (HA), responsible for binding to sialic acid receptors, entry into the cell and fusion of the viral envelop with the endosome; neuraminidase (NA), which removes sialic acids, allowing for viral release from infected cells; and, the ion channel matrix 2 (M2) protein, which is responsible for the acidification of the virion following endocytosis, and viral assembly. Under the viral envelope, there is a protein layer that is made of the matrix 1 (M1) protein, which is involved in virion assembly and budding. The nuclear export protein (NEP) is found inside the viral particle and it is required for the nuclear export of the eight viral ribonucleoprotein (vRNP) complexes from the nucleus to the cytoplasm at the late stages of viral replication. The vRNP complexes, which are present in the core of the virion, are made of the negative-sense, single-stranded viral (v)RNAs packed by the viral nucleoprotein (NP) and the three subunits (PB2, PB1, and PA) of the RNA-dependent RNA polymerase (RdRp) complex that are responsible for viral RNA genome replication and gene transcription in the nuclei of infected cells. IAV proteins and their schematic representation are shown at the bottom. B) Genome organization: The IAV genome is made of eight single-stranded, negative-sense, vRNA segments (PB2, PB1, PA, HA, NP, NA, M, and NS). White boxes represent packaging signals that are responsible for the selective packaging of each vRNA segment into the virion. Numbers represent the nucleotide lengths of each of the 3′ and 5′ packaging signals in each of the vRNAs. Each vRNA is flanked by the 3’and 5´ non-coding regions (NCRs, black lines) recognized by the viral RdRp for viral genome replication and gene transcription.

IAV belongs to the *Orthomyxoviridae* family of enveloped negative sense, single-stranded RNA viruses with a segmented genome [1] (Figure 1). IAVs are classified based on the antigenic properties of the hemagglutinin (HA) and neuraminidase (NA) viral surface glycoproteins into 18 HA (H1 to H18) and 11 NA (N1 to N11) subtypes [2]. Both the HA and NA glycoproteins are the most abundant proteins in the viral envelope, followed by the matrix 2 (M2) protein (Figure 1A) [3]. Under the viral membrane, the inner surface envelope matrix 1 (M1) protein encloses the viral ribonucleoprotein (vRNP) complexes. These vRNPs present the core of the virion and consist of the viral (v)RNA segments that are coated with viral nucleoprotein (NP), and one single copy of the viral heterotrimeric polymerase complex that is made up of the polymerase acidic (PA) and basic 1 and 2 (PB1, PB2) proteins [4,5]. The eight vRNA segments (PB2, PB1, PA, HA, NP, NA, M, and NS) contain long central coding regions that are flanked at both termini by non-coding regions (NCRs) that are critical for vRNA genome replication and gene transcription (Figure 1B) [6]. In the most external 3′ and 5′ ends of each vRNA segment, packaging signals are located and needed for virus assembly [7].

During IAV infection, the binding of the viral HA to host oligosaccharides containing terminal sialic acid initiates the uptake of the virus by endocytosis. Since cell surface sialyloligosaccharides are highly heterogeneous, differential recognition of sialic acid groups by the viral HA is one of the major determinants of viral tropism and host specificity [8,9]. In general, avian viruses preferentially bind to sialic acid that is attached to galactose by an α2,3 linkage, while human influenza viruses preferentially bind the sialic acid attached to galactose by an α2,6 linkage [8].Upon endocytosis, the acidification of the endosomal lumen triggers conformational change of the viral HA that results in the fusion of the viral and endosome membranes [10]. At the same time, the opening of the M2 ion channel protein allows the acidification of the viral core, leading to the dissociation of the vRNPs from M1, and as a result, the vRNPs are delivered into the cytosol of the infected cell. In a process that is mediated by the viral NP, vRNPs are transported to the nucleus where viral replication and transcription take place. IAV NP and the RNA-dependent RNA-polymerase (RdRP) complexes (PB1, PB2 and PA) are the minimal components for viral genome replication and gene transcription [11,12]. IAV is able to transcribe individual (PB2, HA, NA, and NA) or multiple (PB1, PA, M, and NS) proteins from each viral segment [13,14,15,16]. Newly synthesized vRNPs need to be exported from the nucleus to the cytoplasm and then transported to the cell surface in order to be packaged into the new viral particles. In this regard, the assembly of a nuclear export complex consisting of vRNP, M1, and the nuclear export protein (NEP), which contains two nuclear export signals, is required [17,18]. After their synthesis in the endoplasmic reticulum (ER), the structural proteins HA, NA, and M2 are transported to the plasma membrane directed by the apical sorting signals [19]. For successful packaging, the eight vRNA segments are incorporated into nascent virions by the selective packaging signals that are present in each of the vRNAs (Figure 1B) [7,20]. Virion assembly and budding are initiated once all of the viral structural proteins and vRNAs are transported and packaged in the apical surface, [21,22]. For the complete release of the viral particle, the sialidase activity of the NA protein is critical for removing the binding between the HA spikes and the sialic acid receptors in the surface of infected cells [23,24].

### 1.2. Current Vaccine Strategies for the Control of Human Influenza Infections

IAVs cause annually recurrent epidemics that are responsible for mild to severe respiratory illness or even death in humans, which are mainly associated with complications after bacteria secondary infections [25,26,27], representing a threat to public health and global economy [28,29,30].

When new viruses are introduced in the human population IAVs can also cause occasional pandemics of great consequences [31]. Currently, vaccination remains the main and most effective strategy to protect humans against seasonal IAV [32,33]. Although recommendations vary between countries, in the United States (US), the Advisory Committee for Immunization Practices (ACIP) recommends influenza vaccination every year for all persons older than six months [34]. However, because IAVs can rapidly evolve due to the constant antigenic drift, seasonal vaccines need to be reformulated each year to ensure that the HA and NA that were included in the vaccine match the circulating seasonal virus [35]. The World Health Organization (WHO) has the main role in surveillance across the world by using data obtained from more than 130 national influenza centers that are located in over 100 countries to identify the antigenicity and sequence of current circulating viruses and perform the correct update of the vaccine for the following influenza season [36,37].

**Figure 2:**
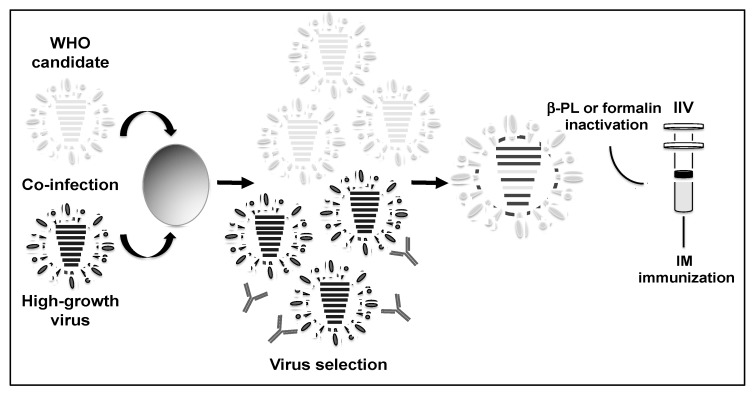
Production of the inactivated influenza vaccine (IIV): To prepare IIV, 10–11 days old chicken embryonated eggs are infected with two IAVs: the candidate virus recommended by the WHO (top, gray) and a high-growth virus (bottom, black). Reassortant viruses are harvested from the allantoic fluid of infected eggs 2–3 days post-infection and the appropriate reassortant virus to be included in the IIV containing the HA and NA viral segments from the WHO candidate virus (gray) and the six internal segments (PB2, PB1, PA, NP, M, and non-structural (NS)) of the high-growth virus (black) is selected by amplification in the presence of antibodies against the HA and NA glycoproteins of the high-growth virus. Genomic composition of the reassortant virus must be confirmed by sequencing. The selected virus to be used in the IIV is chemically inactivated with β-propiolactone (β-PL) or formalin, concentrated, purified for vaccine production, and then administrated intramuscularly (IM).

Three types of vaccines have been approved by the Food and Drug Administration (FDA) for use in the human population: recombinant viral HAs, inactivated influenza vaccine (IIVs), and live attenuated influenza vaccines (LAIVs) [38]. The IIV, which was introduced and licensed in the 1940s, is the most common vaccine for the treatment of influenza virus infections. IIVs are produced by co-infection of embryonated chicken eggs with the strain annually recommended by the WHO (WHO candidate) and a high-growth virus, such as A/Puerto Rico/8/1934 H1N1 (PR8) (Figure 2) [39]. At 2–3 days post-infection, the desired 6:2 reassortant virus, containing the HA and NA viral segments from the WHO recommended the circulating strain and the backbone of the high-grow virus, is selected by amplification in the presence of neutralizing antibodies against the HA and NA of the high-growth virus (Figure 2). Next, the virus is concentrated, purified, and then finally inactivated with formaldehyde or β-propiolactone (Figure 2) [40]. Through this mechanism, the vaccine is made available as a whole chemically disrupted virus or a purified protein formulation, with both being delivered by intramuscular injection (Figure 2) [40,41,42,43]. IIVs have shown to be 60–90% effective in preventing the morbidity and mortality associated with influenza infection [44,45,46] by inducing a strong humoral immune response towards the surface viral glycoproteins HA and NA [47]. However, IIVs induce limited T cell-mediated immune responses and the elicited cellular responses are mainly strain and subtype specific [48,49,50].

**Figure 3:**
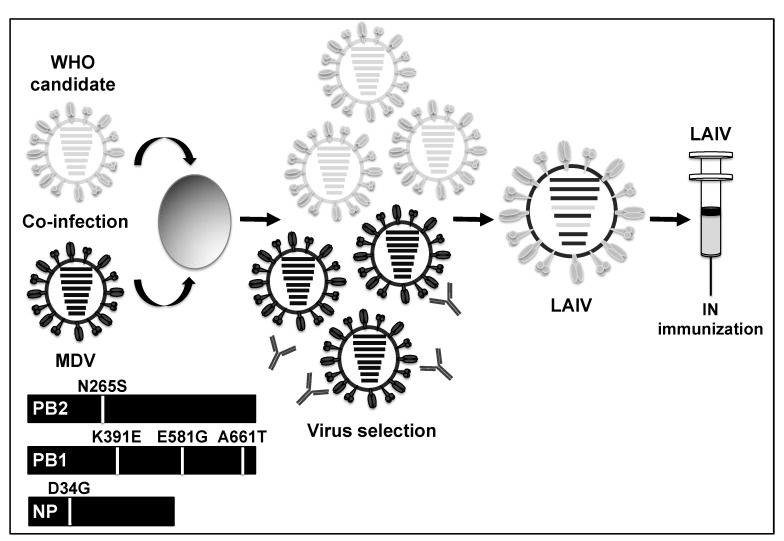
Generation of the live-attenuated influenza vaccine (LAIV): The LAIV is produced by co-infection of 10–11 days old chicken embryonated eggs with the candidate virus recommended by the WHO (top, gray) and the A/Ann Arbor/6/60 H2N2 (bottom, black) Master Donor Virus (MDV). After 2–3 days post-infection, the appropriate reassortant seed virus containing the six internal segments (PB2, PB1, PA, NP, M, and NS) derived from the MDV (black) and the two glycoprotein (HA and NA) segments from the recommended WHO circulating strain (gray) is selected by amplification at low temperatures in the presence of antibodies against the MDV, HA, and NA. The selected LAIV is then administrated intranasally (IN). Amino acid substitutions in the PB2 (N265S), PB1 (K391E, E581G, and A661T) and NP (D34G) viral segments responsible for the attenuated (att), temperature sensitive (ts), and cold-adapted (ca) phenotype of the MDV A/Ann Arbor/6/60 H2N2 are indicated at the bottom.

In contrast to IIVs, LAIVs are intranasally administrated and they mimic the natural route of influenza infection. They induce broader and more robust immune responses that resemble those induced during natural viral infection, including cellular and humoral responses in the mucosal [51,52,53]. Importantly, LAIVs can prime a specific T cell-mediated immune response in naive populations and provide cross-protection against heterosubtypic strains, including pandemic influenza viruses. This LAIV protection profile is a highly desirable requisite for effective influenza vaccine candidates [54,55]. The strategy to produce LAIVs is based on the co-infection of chicken embryonated eggs with the WHO candidate and a Master Donor Virus (MDV) (Figure 3). The segmented nature of the influenza virus genome allows for the generation of a reassortant virus containing the HA and NA of the selected WHO candidate and the six remaining internal segments from the MDV. The 6:2 reassortant virus is selected by passaging in the presence of neutralizing antibodies against the surface glycoproteins of the MDV strain and at low temperature (25 °C) (Figure 3). The MDV that is used in the US to produce LAIVs for IAV is the A/Ann Arbor/6/60 H2N2 [56,57], which was generated after serial passages under gradually low temperatures of the parental A/Ann Arbor/6/60 H2N2 virus in primary chicken kidney (PCK) cells [58]. This process ensures that the selected MDV has a cold adapted (ca), temperature sensitive (ts) and attenuated (att) phenotype, which has been shown to be conferred by four mutations in two of the viral polymerase components PB2 (N265S) and PB1 (K391E, D581G, and A661T) and one in the viral NP (D34G) [59]. As a result, the MDV displays limited replication at high (37 °C or 39 °C) temperatures, but it has efficient replication at lower (25 °C to 33 °C) temperatures [59,60,61]. This temperature restriction allows for the LAIV to efficiently replicate in the colder human upper respiratory tract, while avoiding viral replication in the warmer lower respiratory tract, where influenza virus infection causes diseases [62]. Due to the fact that the ca, ts, and att phenotype of the LAIV is conferred by five mutations in three different viral segments (PB2, PB1, and NP), it is not likely to revert to a virulent form, providing the LAIV with a high safety profile [60]. In any case, each strain that is incorporated into a LAIV is tested to ensure that the ca, ts, and att phenotype has been retained and clinically evaluated to assess its safety before final release for widespread use [63]. In addition, sublingual administration of the LAIV has been evaluated and compared to intranasal administration [64]. Intranasal administration of the LAIV could lead to virus invasion of olfactory bulbs, representing an important safety concern [65]. However, sublingual administration could overcome this risk while also being equally effective in the protection against lethal viral infection [64].

Currently, two IAV subtypes (H1N1 and H3N2) and two influenza B virus (IBV) lineages (Victoria and Yamagata) are co-circulating in humans and they are responsible for seasonal influenza epidemics. The first LAIV (FluMist; MedImmune) that was approved for use in the US was a trivalent vaccine that contained antigens from both IAV H1N1 and H3N2 subtypes and one IBV lineage (either Victoria or Yamagata) [66,67]. This trivalent LAIV was commercialized in 2003 as a nasal spray and was shown to be safe, well tolerated, and highly efficient in both adults and children [68,69]. In 2012, the FDA approved a quadrivalent vaccine formulation containing both IBV Yamagata and Victoria components, in order to provide protection against the two IBV lineages as well as the two IAV subtypes (H1N1 and H3N2) circulating in humans [70]. In the quadrivalent LAIV (Flumist), two cold-adapted MDVs, A/Ann Arbor/6/60 H2N2, and B/Ann Arbor/1/66, are used as backbone to generate four 6 + 2 reassortant viruses with the external HA and NA glycoproteins from the circulating IAVs (H1N1 and H3N2) and IBVs (Victoria and Yamagata lineages), respectively [56,71]. Currently, the quadrivalent’s use has been restricted to healthy people aged 2–49 years of age who are not pregnant, since the mechanism of attenuation has not been completely understood [72,73].

### 1.3. The Need of Novel LAIV Approaches to Combat Influenza Infections

In the past decades, influenza vaccine preparation has evolved to ensure effective protection against seasonal viral infections, while maintaining a good safety profile [38]. The degree of protection after vaccination is known to be dependent on the antigenic match between the vaccine strains and those that are circulating in the community, the age of the vaccine recipient, and the previous history of viral infection and/or vaccination [74,75]. The continuous mutations that IAV undergoes in the viral HA and NA proteins directly affect the Antigenic match, which affects the recognition by vaccine-induced antibodies or by any preexisting immunity [76]. Vaccine effectiveness is annually evaluated mainly using data routinely acquired from individuals with influenza-like illness as an estimated percentage effectiveness adjusted for age, timing, and geography [77,78]. Historically, the LAIV has been shown to provide greater efficacy than the IIV in children, with absolute LAIV efficacy rates of 75–80% [69,79,80]. However, different studies revealed low effectiveness among children of the quadrivalent LAIV during the 2013–2014 (18%), 2014–2015 (28%), and 2015–2016 (48%) seasons [81,82,83,84,85]. Based on this low effectiveness, the ACIP concluded that a preference for the quadrivalent LAIV over the IIV was no longer warranted and then recommended that the quadrivalent LAIV should not be used during the subsequent 2016–2017 and 2017–2018 influenza seasons [34,86]. However, for the 2018–2019 influenza season, the use of the LAIV has again been a recommended option, but with no preference over the IIV [87].

To date, the real cause(s) of reduced effectiveness of the quadrivalent LAIV are not known, but several factors have been suggested to contribute to the lower efficacy of the LAIV [77,88,89]: (1) pre-existing immunity in the adult population could suppress local mucosa replication of the LAIV that is required for the induction of an effective protective immune response [90]; (2) reduced viral infection of the IAV H1N1 component of the LAIV because of reduced sialic acid binding of the not fully human-adapted viral HA as compared to the H3N2 and/or IBV LAIV components [91,92]; (3) the acquisition of mutation(s) in the M segment of the MDV A/Ann Arbor/6/60 H2N2 that affects the pH stability of the vaccine, having a higher impact on the IAV H1N1 component of the LAIV [93]; (4) the introduction of a second IBV component in the quadrivalent LAIV formulation that could interfere with the replication of the H1N1 component of the LAIV [94]; (5) heat stability, since the H1N1 component of the LAIV that was used during the 2013–2014 seasonal vaccine had increased susceptibility to thermal degradation [95]; and, (6) a high-content of defective-interfering (DI) viral particles in the LAIV that compromise virus replication in the respiratory tract, leading to a decrease in the immune response to the vaccine [96].

Although the reason(s) of the reduced effectiveness of the LAIV has not been fully understood and multiple variables could be responsible for its low efficiency, the LAIV has several advantages over the IIV for the control of influenza viral infections. These include, among others, the intranasal route of administration of LAIV over the intramuscular administration of the IIV [97] and the ability of LAIV to induce more robust and long-term humoral and cellular immune responses than IIV [98]. For developing countries where a limited number of health care workers are available to administrate the vaccine in a short period of time, LAIVs are especially attractive because of the use of a single-dose and needle-free delivery system [99,100]. A recent phase IV clinical trial has also evaluated the feasibility of Flumist LAIV self-administration and demonstrates equally safe and immunogenic profile than when health care workers administer the LAIV [101]. Therefore, new approaches are still urgently needed to develop and guarantee the ideal LAIV profile, which includes safety, efficacy, stability, ease of administration, and production for the treatment of seasonal and potential pandemic influenza viral infections in humans.

### 1.4. Reverse Genetics Techniques for the Development of Novel Influenza Vaccines

Because of the changing nature of influenza virus, vaccine production to develop high-yield strains as vaccine components is a constant challenge. Manufacturing influenza vaccines usually take around four to five months to produce, package, test, and release for use in the upcoming season [63]. The implementation of reverse genetics approaches into research has advanced our understanding of the biology of influenza virus, including the mechanisms that are involved in viral genome replication and gene transcription, pathogenesis, or viral-host interactions [102,103,104,105]. Reverse genetics strategies have also enabled the modification of the influenza viral genome to generate recombinant viruses expressing foreign proteins as vaccine vectors [106,107], harboring reporter genes to easily track viral infections [106,108,109,110], or carrying pre-determined mutations that result in viral attenuation for their potential implementation as safe, immunogenic, and protective LAIVs [99,111,112,113].

**Figure 4:**
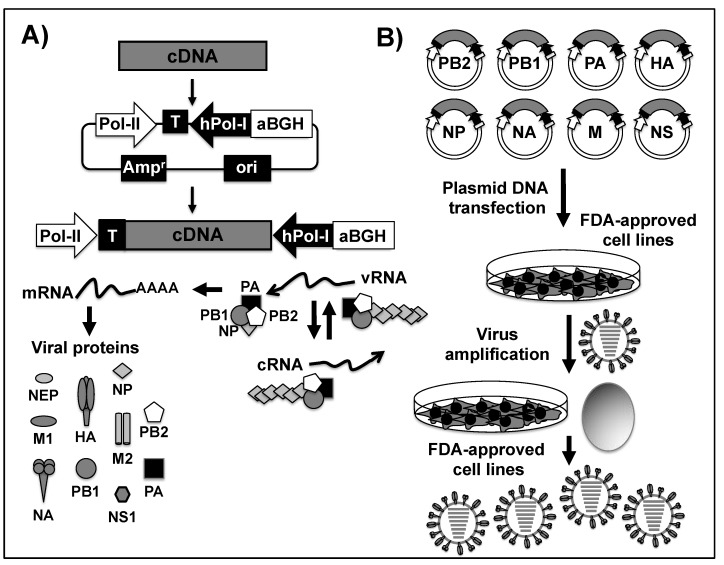
IAV vRNA cloning into ambisense/bidirectional plasmids for the generation of recombinant viruses using plasmid-based reverse genetic approaches. (**A**) Schematic representation of the ambisense/bidirectional rescue plasmid to generate recombinant IAV: Ambisense/bidirectional rescue plasmids containing the human polymerase I promoter (hPol-I, black arrow) and the mouse polymerase I terminator (T, black box) sequences that regulate the synthesis of the negative sense vRNAs are indicated. In the opposite direction to the Pol-I cassette, and from the same cDNA, the polymerase II dependent promoter (Pol-II, white arrow) and the bovine growth hormone polyadenylation termination sequence (aBGH, white box) direct the synthesis of positive sense mRNA to produce viral proteins. Newly synthesized vRNAs generated from the Pol-I cassette are recognized by the viral RdRp subunits (PB2, PB1 and PA) that, together with the viral NP, lead to the formation of vRNP complexes responsible of viral genome replication and gene transcription. Transcription from newly synthesized vRNAs results in mRNA expression and the production of new viral proteins. Replication of newly synthesized vRNAs results in the formation of complementary (c)RNAs for the amplification and synthesis of new vRNAs that will be incorporated into the nascent virions as novel vRNP complexes. (**B**) Plasmid-based reverse genetics to generate recombinant IAV: FDA-approved cell lines for vaccine production are co-transfected with the eight (PB2, PB1, PA, HA, NP, NA, M, and NS) ambisense/bidirectional IAV plasmids. Viable virus recovered from the tissue culture supernatants 3–4 days after transfection is amplified using fresh FDA-approved cell lines or 10–11 day-old embryonated chicken eggs.

Several plasmid-based reverse genetics approaches have been developed to generate recombinant influenza viruses [114]. Currently, the most common method delivers eight ambisense and bidirectional plasmids for the simultaneous expression of the positive-stranded viral mRNAs and the negative-stranded vRNA in susceptible cells (Figure 4) [104]. This reverse genetics system is based on a dual promoter concept in which a complementary (c)DNA copy of the vRNA is cloned in an ambisense plasmid carrying a RNA polymerase I (Pol I) promoter to direct the synthesis of vRNAs; and, in the opposite orientation, a RNA polymerase II (Pol II) promoter driving mRNA expression from the same cDNA (Figure 4A) [114,115,116]. The eight-ambisense plasmids are then co-transfected into FDA-approved cells and 3–4 days after transfection, viable virus can be recovered from the tissue culture supernatant of transfected cells and then amplified in either fresh FDA-approved cells or chicken embryonic eggs (Figure 4B) [104]. For research and proof-of-concept experiments, human 293T cells, 293T/Madin Darby canine kidney (MDCK) cell mixtures, and African green monkey kidney (Vero) cells have been used [105]. However, when the rescued virus is intended for use in the development of human vaccines, specific regulatory requirements are imposed [117]. Currently, the generation of recombinant viruses for the development of influenza vaccines for human use in the US and Europe is limited to a selected number of mammalian cells, such as MDCK or Vero cells [118,119,120,121,122].

Although the FDA approved the cell-based production method of influenza vaccines in 2012, the global-scale infrastructure for manufacturing the required amount of cell-based influenza vaccines does not currently exist [123]. In fact, the majority of influenza vaccines that are currently commercialized by licensed manufactures are grown in chicken embryonated eggs [124]. One of the reasons why the traditional method for influenza vaccine production by co-infection of using chicken embryonated eggs (Figure 2 and Figure 3) is preferred over reverse genetics approaches is because it allows for the natural selection of a virus variant with a gene constellation that improves egg growth properties, a crucial aspect for the vaccine manufacturing process [40,125]. Unfortunately, most current influenza viruses do not grow well in eggs and this affects the efficiency of the traditional reassortment method. Therefore, influenza plasmid-based reverse genetics (Figure 4) represent a better, faster, and flexible alternative, allowing the rapid generation of virus containing the six internal segments of a high growth PR8 (IIV) or A/Ann Arbor/6/60 H2N2 (LAIV) and the two surface HA and NA glycoprotein segments from the WHO candidate [40,126].

Importantly, due to biosafety concerns and the inherent high lethality in chicken embryos, the classical approach of generating vaccine candidates cannot be used to produce vaccines for highly pathogenic IAV (HPAIV), such H5N1 [36]. This fact has increased the interest of the research community in optimizing reverse genetics methods that would allow the attenuation of H5 or other HPAIV [127,128]. Reverse genetic approaches represent an excellent alternative to the traditional method for influenza vaccine production and it might become the method of choice for developing candidate vaccine in global efforts that are directed towards pandemic preparedness [114].

## 2. New Strategies to Develop LAIVs

During the last few decades, considerable improvements have been accomplished in the development of LAIVs. However, vaccine manufacturing is still a slow process that allows for the emergence of mutations of the selected IAV while the vaccine is being manufactured, leading to less effectiveness than expected [129]. Thus, there is an urgent need for novel approaches to increase the effectiveness of both seasonal and pandemic LAIVs. In the past few years, numerous strategies for the generation of LAIV candidates have been developed using reverse techniques approaches by different research groups. Many of the strategies have provided promising candidates that could be tested and implemented as LAIVs in the near future, and we will discuss the most promising in this review.

### 2.1. NS1 Truncated or Deficient Viruses as LAIVs

**Figure 5:**
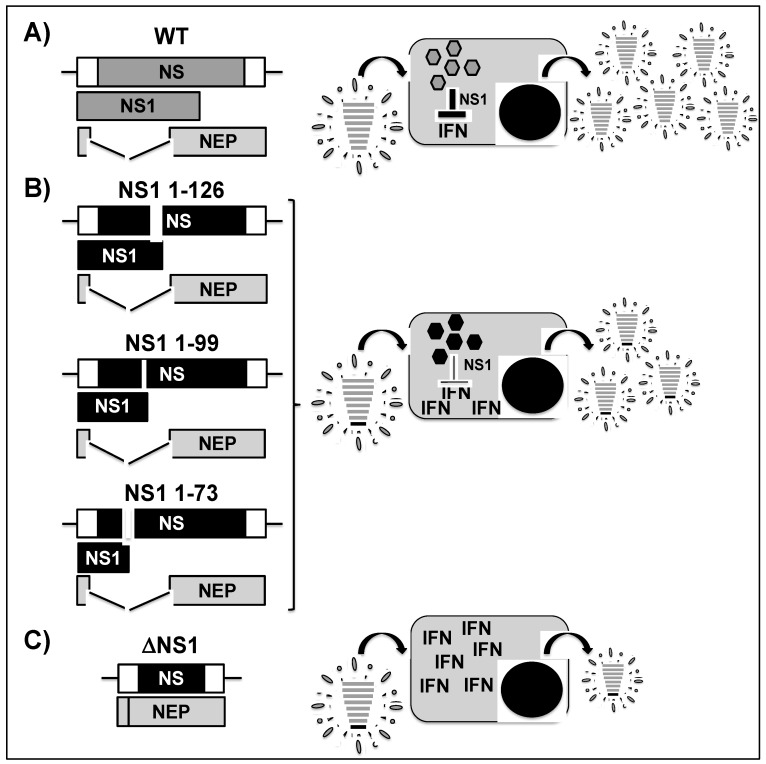
LAIV based on truncations and/or deletion of the viral NS1: Schematic representation of wild-type, WT (**A**), NS1 truncated (**B**), or NS1 deficient (**C**) recombinant IAV. WT NS vRNA is represented in gray boxes. WT NS1 and NEP open reading frames (ORFs) are represented as dark and light gray boxes, respectively. Modified NS segments and truncated NS1 ORFs are indicated in black boxes. White lines represent stop codons. White boxes indicate the packaging signals located at the 3´and 5´ ends of the NS vRNA. Lines at the end of the NS vRNA indicate the 3´and 5´ NCR. Expression of WT NS1 protein (**A**) results in inhibition of interferon (IFN) induction and efficient viral replication. NS1 1–126 (top), 1–99 (middle), or 1–73 (bottom) truncations in the NS1 ORF (**B**) or deletion of the entire NS1 ORF (**C**) result in the higher induction of IFN and reduced levels of viral replication and, therefore, virus production.

The IAV non-structural 1 (NS1) protein is expressed early in infected host cells and it plays multiple roles during the life cycle of the virus [130]. NS1 is the main viral protein that is responsible for counteracting the antiviral response and it acts as an interferon (IFN) antagonist to suppress type I IFN production while promoting viral replication [131,132,133,134,135,136,137,138,139,140,141]. Hence, recombinant IAVs with modified, truncated, or absent NS1 are likely to be reasonable alternatives to generate LAIVs, since they are attenuated in IFN-competent hosts [138,142,143,144]. NS1 is produced from the eight viral segment (NS), which encodes for two proteins through alternative splicing [145] (Figure 5). The primary transcript that is encoded by the IAV NS segment is NS1 and a weak 5´ splice site results in the expression of NEP, which is essential for the nuclear export of vRNAs and viral morphogenesis [14] (Figure 5A). Multiple studies have reported the generation of recombinant equine [146], swine [147,148,149,150], avian [151,152,153,154,155], canine [138], and human [156,157,158] IAVs with partial truncations or absent NS1, which are highly attenuated in IFN-competent hosts. This strategy has proven to be immunogenic and protective in different species, such as mice [138,157,159], horses [146], pigs [147,148,149,150], birds [151,152,153], and macaques [156]. Therefore, NS1 mutant IAVs seem to be an excellent option for implementation as safe, immunogenic, and protective LAIVs for the treatment of multiple IAVs in different animal hosts. In fact, the safety and immunogenicity of a trivalent vaccine formulation containing NS1 deficient IAV H1N1 and H3N2 and IBV have been evaluated in phase I/II human clinical trials [160]. The vaccine was shown to be safe and able to induce significant levels of antibodies in healthy adult (ClinicalTrials.gov identifier NCT01369862).

A correlation between the length of the NS1 protein and IAV attenuation in vivo has been reported [148,151,159,161]. For instance, Nogales et al., have recently described the safety, immunogenicity, and effectiveness of LAIV candidates containing C-terminal truncations (NS1 1–126, NS1 1–99, and NS1 1–73) (Figure 5B) or an entire deletion (ΔNS1) (Figure 5C) of the viral NS1 protein to protect against H3N8 canine influenza virus (CIV) [138]. Interestingly, in vitro, these viruses replicated at similar levels as the wild-type (WT) H3N8 CIV at 33 °C, representing great advantage in vaccine production. Moreover, the authors showed that recombinant CIVs encoding truncated (Figure 5B) or deleted (Figure 5C) NS1 proteins had reduced ability to counteract IFN activation compared to the WT H3N8 CIV (Figure 5A). Notably, NS1 mutations resulted in the attenuation of the virus in vivo in a mouse model of influenza infection and ex vivo in canine tracheal explants, with the ΔNS1 virus showing the greatest degree of attenuation [138]. In addition, mice that were vaccinated with a single intranasal dose of modified NS1 viruses were protected against challenge with WT H3N8 CIV. Most likely, the ability of these viruses with altered NS1 proteins to induce an IFN response, which is usually blocked during the WT viral infection, resulted in an optimal balance of attenuation and protective immunogenicity: the desired outcome for a safe and effective LAIV [162,163].

### 2.2. Suboptimal Codon Usage for the Development of LAIV

Due to the degeneracy of the genetic code, most amino acids are encoded by more than one codon, termed as synonymous codons [164]. Codon usage bias refers to the unequal frequency in the use of synonymous codons between and within species [164,165]. The existence of preferred codons can affect the levels of gene expression, protein translation, and protein folding [164,166,167,168,169,170,171]. Usually, the codon usage of viruses, including IAV, is similar to that of the host that they infect so that the translation is the most efficient. In the case of pandemic IAVs carrying avian or swine origin segments, an adaptation of the virus codon use might be expected to reflect human codon use. However, although human codon usage imposes certain selection pressure on the avian or swine viruses, it has been shown that human genes and most viral genes from pandemic IAV strains had a negative correlation in codon usage [168,169]. In this regard, two models have been proposed to explain viral codon usage: the translation or selection model is based on the adaptation to the host transfer (t)RNA abundance as a strategy to improve viral fitness [169]; and, the mutational or neutral model is based on certain mutations in GC-rich regions can provide advantages to the virus to escape from the host antiviral response [164,166,168,169,172]. Interestingly, human seasonal IAV strains that were isolated in recent years have been shown to have a reduced GC content in their genome, which could be advantageous in escaping immunity [168].

By taking advantage of virus and host codon usage and new technologies that allow for *de novo* synthesis of modified viral genes, researchers have implemented a new concept for the development of LAIVs by using suboptimal codon usage in viral proteins [173,174,175,176,177,178]. Codon deoptimization refers to the use of misrepresented codons in the viral genome to generate attenuated viruses [172,179,180]. This strategy has been implemented as a mechanism of viral attenuation by incorporating hundreds of synonymous mutations into single or multiple IAV genes [175]. The use of codon deoptimized IAVs has several advantages over other strategies for their implementation as LAIV: i) they could be synthesized in a few weeks for any emerging IAV once its genome is known; ii) deoptimized IAVs contain the intact WT amino acid sequence and they therefore express an identical antigenic repertoire of T- and B-cell epitopes; iii) due to the large number of nucleotide changes introduced in one or several viral genes, the probability of reversion to a virulent WT phenotype is extremely unlikely, if possible at all; and, iv) they can efficiently replicate in vitro, important for vaccine production, while being highly attenuated in vivo, important for their safe implementation as LAIV [174,177,178].

IAVs circulating in different hosts have been found to have distinct codon usage patterns, which may reflect host adaptation [168]. Fan et al. have described that it is feasible to generate a human seasonal IAV that is specifically attenuated in human cells, but not in eggs, by converting its codon usage to that present in avian IAVs [176]. In this work, the authors introduced over 300 mutations into the genome of the seasonal A/Brisbane/59/2007 H1N1 [176]. Corroborating the hypothesis of the authors, the resultant virus was attenuated in mammal cells and mice, being important for its safe implementation as an LAIV, while displaying efficient growth in eggs, which is important for vaccine production. Remarkably, in mice, this virus elicited a strong immune response and conferred protection against both homologous and heterologous IAV challenges. They also demonstrated the potential use as LAIV of the deoptimized H1N1 IAV containing the HA and NA from other IAVs, suggesting that it could be used as new MDV to generate future LAIVs [176].

**Figure 6:**
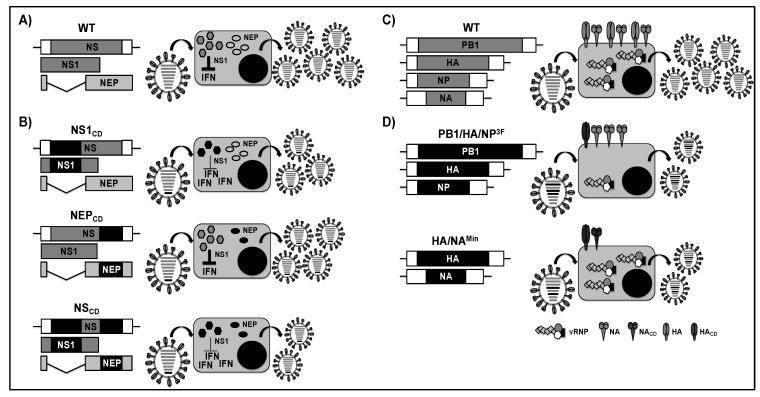
Codon deoptimization (CD) for the generation of LAIV: (**A**,**B**) Schematic representation of WT (**A**) and CD (**B**) viral NS segments: WT NS vRNA is represented in gray boxes (**A**). WT NS1 and NEP ORFs are represented as dark and light gray boxes, respectively (**A**). CD NS1 (NS1_CD_, top), NEP (NEP_CD_, middle) or both NS1 and NEP (NS_CD_, bottom) proteins as well as their respective NS vRNA segments are indicated with black boxes (**B**). After infection with a virus encoding a WT NS segment, expression of NS1 results in inhibition of IFN induction, allowing the efficient viral replication (**A**). Infection with viruses encoding a codon deoptimized NS1 protein (NS1_CD_, top; NS_CD_, bottom) results in reduced NS1 protein expression levels and inefficient inhibition of type I IFN responses, resulting in reduced viral replication and viral production. CD of NEP (NEP_CD_, middle) results in lower expression of NEP, without significantly affecting viral replication. CD of NS1 and NEP (NS_CD,_ bottom) results in higher attenuation than viruses containing only NS1 or NEP CD ORFs, correlating with the amount of codon changes introduced in the viral segment. (**C**,**D**) Schematic representation of WT and IAV attenuated by codon-pair bias: WT PB1, HA, NP, and NA vRNA segments are indicated in gray boxes (**C**). Codon-pair deoptimized PB1, HA, and NP (PB1/HA/NP^3F^) (**D**, top); or, HA and NA (HA/NA^Min^) (**D**, bottom) proteins are represented in black boxes. During WT viral infection (**C**), vRNPs mediate viral genome replication and gene transcription, allowing efficient viral protein synthesis and viral production. Likewise, optimal expression of the viral HA and NA results in efficient production of infectious viral progeny. Infection with PB1/HA/NP^3F^ virus (**D**, top), results in reduced levels of viral replication and transcription mediated by lower levels of PB1 and NP expression. The codon-pair deoptimization of HA also affects protein expression levels, contributing to the attenuation of the PB1/HA/NP^3F^ virus in mice but with reasonable growth in vitro. Likewise, the reduction in the level of expression of the viral HA and NA during infection with the HA/NA^Min^ virus (**D**, bottom) results in reduced virion formation and therefore lower infectious viral production. White boxes (**A**–**D**) indicate the packaging signals that were located at the 3´and 5´ ends of each of the vRNAs. Lines at the end of each vRNA (**A**–**D**) indicate the 3´and 5´ NCR.

The NS segment has also been targeted by codon deoptimization for the generation of LAIV (Figure 6A,B) [178]. Three codon deoptimized recombinant PR8 viruses were generated containing silent mutations in the coding regions of NS1 (NS1_CD_), NEP (NEP_CD_), or both (NS_CD_) (Figure 6B). Although the three viruses grew to similar levels as WT PR8 virus in MDCK cells, the replication capabilities of NS_CD_ and NS1_CD_ were impaired in human lungs epithelia carcinoma A549 cells, which was likely due to differences in the IFN responses between the A549 and MDCK cells [181]. In fact, bioassays to evaluate the production of IFN showed that NS_CD_ and NS1_CD_ viruses (Figure 6B) counteracted the IFN response to a lesser extent than the WT PR8 virus (Figure 6A). Likewise, NS_CD_ and NS1_CD_ viruses were significantly attenuated in mice when compared to the WT virus, while NEP_CD_ similarly replicated WT PR8. Those data suggested that a correlation exists between the extent of deoptimization and levels of viral attenuation, since the viruses with more codon deoptimized substitutions were more attenuated (NS_CD_ > NS1_CD_ > NEP_CD_). The authors also showed that a single-dose intranasal immunization with NS_CD_ was sufficient to induce a strong humoral response in mice and to protect animals against homologous and heterologous lethal challenges, providing an effective viral segment target for codon deoptimization to develop LAIV candidates.

Codon-pair bias refers to the fact that some pairs of codons occur more frequently than others and this frequency differs between species [182,183]. The codon-pair bias approach has also been used to generate LAIV using codon combinations that are less represented in the genetic code of the host and to alter the expression of viral proteins involved in the synthesis of vRNA and viral spread [173,174]. Mueller et al. used the Synthetic Attenuated Virus Engineering (SAVE) approach to recode and synthesize the viral genome of PR8 in a way that preserved the WT amino acid sequence but created a suboptimal arrangement of codon pairs [173]. They modified the viral PB1, NP, and HA genes of WT PR8 (Figure 6C) to generate recombinant viruses containing one deoptimized segment (PB1^Min^, NP^Min^ or HA^Min^) or a combination of three deoptimized segments (PB1/NP/HA^3F^) (Figure 6D, top). The authors did not find significant differences in the growth kinetics or plaque phenotype of mutant deoptimized viruses when compared to WT PR8. However, in all of the mutant viruses, protein expression of the deoptimized viral gene product was specifically reduced when compared to the same protein in the WT-infected cells. In vivo analysis showed that all of the deoptimized viruses were remarkably attenuated in mice, especially PB1/NP/HA^3F^, which led to a cumulative attenuation of 13,000 fold over WT PR8. Importantly, a single intranasal immunization of mice induced specific immunity that protected them against the homologous WT PR8 challenge. However, these studies did not evaluate heterologous protection.

Codon-pair bias refers to the fact that some pairs of codons occur more frequently than others and this frequency differs between species [182,183]. The codon-pair bias approach has also been used to generate LAIV using codon combinations that are less represented in the genetic code of the host and to alter the expression of viral proteins involved in the synthesis of vRNA and viral spread [173,174]. Mueller et al. used the Synthetic Attenuated Virus Engineering (SAVE) approach to recode and synthesize the viral genome of PR8 in a way that preserved the WT amino acid sequence but created a suboptimal arrangement of codon pairs [173]. They modified the viral PB1, NP, and HA genes of WT PR8 (Figure 6C) to generate recombinant viruses containing one deoptimized segment (PB1^Min^, NP^Min^ or HA^Min^) or a combination of three deoptimized segments (PB1/NP/HA^3F^) (Figure 6D, top). The authors did not find significant differences in the growth kinetics or plaque phenotype of mutant deoptimized viruses when compared to WT PR8. However, in all of the mutant viruses, protein expression of the deoptimized viral gene product was specifically reduced when compared to the same protein in the WT-infected cells. In vivo analysis showed that all of the deoptimized viruses were remarkably attenuated in mice, especially PB1/NP/HA^3F^, which led to a cumulative attenuation of 13,000 fold over WT PR8. Importantly, a single intranasal immunization of mice induced specific immunity that protected them against the homologous WT PR8 challenge. However, these studies did not evaluate heterologous protection.

More recently, a similar approach was used by Yang et al. [174] and Broadbent et al. [177] to generate recombinant PR8 and A/California/7/2009 pandemic H1N1 (pH1N1) viruses, respectively, where the codon pairs in HA (PR8-HA^Min^), NA (PR8-NA^Min^) [174], or HA and NA (PR8 or pH1N1-HA/NA^Min^) [174,177] were deoptimized (Figure 6D, bottom). In both studies, all of the variants (PR8 and pH1N1) were efficiently replicated in MDCK cells, although a reduction in HA and/or NA protein expression was observed (Figure 6D, bottom). PR8-HA/NA^Min^ was evaluated in mice and it displayed severe attenuation while protecting against homologous PR8 and heterologous (A/Aichi/2/68 H3N2 or A/Victoria/3/75 H3N2) viral challenges, which is likely due to the induction of both humoral and cellular immune responses [174]. Likewise, attenuation, immunogenicity, and protection efficacy of pH1N1-HA/NA^Min^ were evaluated in ferrets [177]. Although this virus was attenuated, it was able to induce a humoral immune response that protected against homologous challenge. In summary, both of the authors demonstrated that, although the expression of HA and NA glycoproteins is reduced by deoptimization of codon pairs, modified viruses are still immunogenic and able to induce a protective immune response. Likely, a combination or individual use of either strategy—codon usage and codon-pair bias—might be considered to be a feasible approach for the development of safe, immunogenic, and protective LAIV in the future.

### 2.3. Viral Genome Rearrangement for the Development of LAIVs

IAVs carrying rearranged genomes have shown to serve as LAIV candidates offering several advantages over other LAIV approaches: (i) because of the reorganization of the genome, it is unlikely, if not impossible, for the virus to revert to WT; (ii) foreign genes could be expressed by altering the viral genome organization without altering protein expression; (iii) the reorganization of the viral genome does not affect the antigenicity of viral proteins; and, (iv) rearranged viruses expressing additional HA and/or NA could be used as bivalent LAIVs to protect against multiple strains.

**Figure 7:**
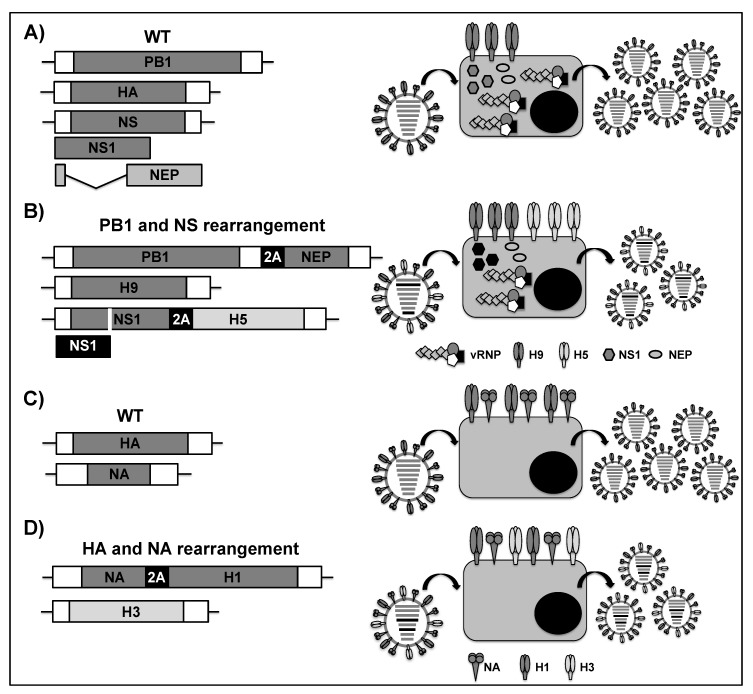
Rearrangement of IAV genome for the generation of LAIV: (**A**,**B**) Schematic representation of WT (A) and rearranged viral segments 2 (PB1) and 8 (NS) (B): WT segments 2, 4, and 8 (A) or rearranged segment 2 (PB1 and NEP) and segment 8 (NS1) are indicated with dark gray boxes (B) The light gray box indicates the secondary H5 inserted in the NS segment. White boxes (A and B) indicate the packaging signals located at the 3´and 5´ ends of each vRNA. Black boxes (B) indicate the sequence of the foot-and-mouth disease virus (FMDV) 2A autoproteolytic cleavage site. Lines at the end of each vRNA (A and B) indicate the 3´and 5´ NCR. A white line in the NS segment (B) represents a stop codon in the NS1 resulting in a truncated (1-99 amino acids) NS1 protein (black box). Expression of NEP from a single polypeptide downstream of the modified PB1 viral segment results in a reduction on the activity of the PB1and an impaired growth of the rearranged virus (B). The expression of the H5 ORF from a modified segment 8 results in an LAIV expressing two different HA (H9, dark gray; and H5, light gray) and the induction of neutralizing antibodies against the two viral glycoproteins. (**C**,**D**) Schematic representation of WT (C) and rearranged segment 4 (HA) and segment 6 (NA) viruses (D): WT (C) and rearranged segment 4 expressing NA-HA (D) are represented in dark gray boxes. Rearranged segment 6 expressing a secondary HA is represented in a light gray box (D). White boxes indicate the packaging signals located at the 3´and 5´ ends of each vRNA (C and D). Black boxes indicate the sequence of the porcine teschovirus (PTV-1)2A autoproteolytic cleavage site (D). Lines at the end of each vRNA indicate the 3´and 5´ NCRs (C and D). While WT virus expresses the HA and NA glycoproteins from the segment 4 and 6 respectively (C), the rearranged virus expresses both the subtype 1 HA and NA glycoproteins from the modified segment 4; and the subtype 3 HA from the modified segment 6, where NA is normally located (D). This rearrangement of the viral genome results in an attenuated recombinant virus able to induce neutralizing antibodies against the two viral glycoproteins (H1 and H3) (D).

The genome rearrangement strategy has been used for developing LAIVs against H9N2 and H5N1 HPAIV, which carry pandemic potential [184,185]. To that end, A/Guinea fowl/Hong Kong/WF10/1999 H9N2 was modified to express a second HA from A/Vietnam/120320/04 H5N1 [186]. The authors employed the viral NS segment for the stable expression of H9N2 NS1 and H5N1 HA protein, which were separated using the foot-and-mouth disease virus (FMDV) 2A autocleavage site, allowing for individual expression of both viral proteins (Figure 7A,B) from the same viral transcript. The NS segment was also modified so that it encoded a truncated NS1 (1–99) [159] and the viral NEP was expressed in segment 2 (PB1) using another FMDV 2A autocleavage site (Figure 7B). This genome arrangement strongly attenuated the virus expressing both the H9 and H5 proteins in vivo. When the H9N2-H5 virus was administrated as a LAIV into mice and ferrets, it provided protection against a lethal challenge with A/Vietnam/1203/2004 H5N1 and a pH1N1 reassortant virus expressing H9 [186].

More recently, Harding et al. generated a PR8 virus containing two HAs (H1 and H3) [187]. The authors modified segment 4 (HA) of WT PR8 (Figure 7C) to encode PR8 NA, followed by a furin cleavage site and the porcine teschovirus 1 (PTV1) 2A cleavage site, allowing for co-linear expression but individual translation of both viral proteins (Figure 7D). Likewise, segment 6, where NA is encoded (Figure 7C), was modified to encode a different viral HA from an H3 virus (Figure 7D). First, they assayed the ability of this technology to allow for the incorporation of both H1 and H3 subtypes in the same virion by using the HA from A/Hong Kong/1968 H3N2. Importantly, dual-HA H1/H3 virions packaged similar levels of both the H1 and H3 glycoproteins as the single HA parent PR8 virus. However, a delay was observed in the growth kinetics profile of the dual-HA when compared to the WT PR8, suggesting that the increased genome size could lead to viral attenuation. This dual-HA H1/H3 virus was attenuated in mice and it elicited high levels of neutralizing antibodies that were able to neutralize virus at similar levels as those elicited from the parental PR8 infection. Vaccination with the inactivated dual-HA virus fully protected mice from challenge that are associated with either PR8 or X31, demonstrating that those antibodies generated after dual-HA H1/H3 virus vaccination were protective.

Although the overall aim of the authors in this study was to develop a platform to allow for the efficient growth of influenza H3 subtypes in eggs, the authors also generated viruses carrying HA segments from currently circulating IAV vaccine strains (H1N1 and H3N2), as well as IBV from Victoria and Yamagata lineages. They observed robust growth for all of the recombinant viruses, indicating that there was no functional interference between the two HA proteins, with no emerging mutations being observed after several rounds of growth in eggs. Finally, they evaluated the antigenic stability of an H3 protein that is normally unstable during growth in embryonated chicken eggs (A/Fujian/411/2002 H3N2) [188]. The coexistence of this H3 protein with H1 PR8 in the virion lead to an increase (~4 orders of magnitude) in growth titer in embryonated chicken eggs as compared to the standard 6:2 reassortant in the PR8 background with the glycoproteins of A/Fujian/411/2002 H3N2. In addition, no mutations in HA were observed after passaging in eggs, suggesting that this approach allows for antigenically stable growth of essentially any clinically relevant IAV strain. Overall, the authors showed that this rearrangement technology is compatible with current vaccine production practices and it can be used to allow the rapid production of more effective LAIVs. However, further studies should be performed to assess not only the stability and immunogenicity, but also the dual protection efficacy of all vaccine candidates for their safe implementation as immunogenic and protective influenza vaccines.

### 2.4. LAIVs Based on the Modification of the Viral M and NS Segments

**Figure 8:**
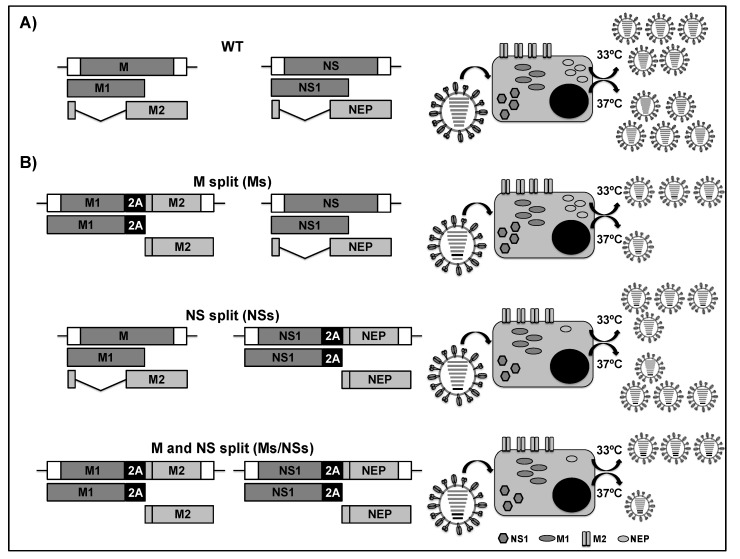
Development of LAIV based on modification of the spliced viral RNA segments 7 (M) and 8 (NS): Schematic representation of WT (A) and modified (B) viral RNA segments 7 (Ms, top), 8 (NS, middle), or both 7 and 8 (Ms/NSs, bottom) in which the overlapping ORFs of the M1 and M2 proteins (Ms, top), NS1 and NEP proteins (NSs, middle), or both (Ms/NSs, bottom) are produced from the same transcript by using the PTV-1 2A autoproteolytic cleavage site. Viral products from the M (M1 and M2) and NS (NS1 and NEP) WT (**A**) or modified (**B**) viral segments are indicated in grey boxes. M2 ORF is shown as a lighter grey box. Black boxes (**B**) indicate the sequence of the PTV-1 2A autoproteolytic cleavage site. The packaging signals located at the 3´and 5´ ends of each vRNA are indicated with white boxes (**A**,**B**). Lines at the end of each vRNA indicate the 3´and 5´ NCR in the M and NS viral segments (**A**,**B**). During infection with WT virus, the optimal expression of M1 and M2 proteins (M segment), as well as NS1 and NEP (NS segment) from the spliced vRNA segments, results in efficient virus replication and production. Infection with a modified M segment (**B**, top) results in slightly reduction of virus replication and production at 33 °C; and significant reduction of viral production at high temperatures (37 °C or 39 °C). Modification of the NS vRNA segment (**B**, middle) results in a slight reduction in virus replication and production that is not temperature dependent. The recombinant virus containing both modified M and NS segments (Ms/NSs) (**B**, bottom) results in impaired viral replication and production, similar to the recombinant virus containing the modified M segment (Ms) at non permissive temperatures (37 °C or 39 °C).

Both the IAV M and NS segments encode two polypeptides using an alternative splicing mechanism: M1 and M2; and, NS1 and NEP, respectively (Figure 8A) [13,14]. Three reorganized PR8 viruses were constructed by Nogales et al. by splitting the overlapping open reading frames (ORFs) of the M1 and M2 (M split; Ms) (Figure 8B, top); the NS1 and NEP (NS split; NSs ) (Figure 8B, middle); or, both (M and NS split; Ms/NSs) (Figure 8B, bottom), using the PTV-1 2A autoproteolytic cleavage site and duplicating the overlapping regions in both viral segments [189]. When evaluating growth kinetics properties in MDCK cells at 33 °C, 37 °C, and 39 °C, the authors found that the NS virus grew at slightly lower titers than WT PR8. However, viruses containing the modified M segment (Ms and Ms/Ns) were severely impaired in replication at 37 °C and 39 °C, whereas high viral titers could be reached at 33 °C, suggesting that the M split segment was responsible for the ts phenotype. Importantly, PR8 viruses that contained the M split segment (Ms and Ms/Ns) were highly attenuated in vivo and they protected mice from a lethal homologous challenge with WT PR8 [189]. Notably, the LAIV candidates that were based on the reorganized M segment (Ms and Ms/Ns) were both more effective in inducing immunity and protecting mice against a lethal homologous challenge with PR8 WT than a PR8 LAIV containing the ts, ca, and att mutations (PB2 N265S; PB1 K391E, D581G, and A661T; NP D34G) of the MDV A/Ann Arbor/6/60 H2N2 used in the human LAIV [59,61,189]. These results showed that the rearrangement of the IAV M segment alone or in combination with the NS segment could be used as a feasible alternative for the generation of safe, immunogenic, and protective LAIV for the treatment of IAV infections.

### 2.5. Single-Cycle Infectious IAVs (sciIAVs) as LAIVs

SciIAVs can be generated by the mutation, deletion, or substitution of viral components using molecular biology techniques. These viruses can be defective in viral genome synthesis, assembly, or release of viral particles, and thus lack the ability to spread after initial infection [190]. Importantly, to generate and propagate sciIAVs, the missing gene product needs to be provided *in trans* in complementing cells lines to support viral propagation [106,191,192]. In non-complementing cells lines, primary infection occurs, but no infectious viral progeny will be produced. For in vivo studies, animals expressing complementing proteins in tissues could be used to allow for infection and progeny production of sciIAVs. In this way, transgenic mice infected with a sciIAV could show pathogenesis but they should not be able to transmit infection to WT mice [193].

**Figure 9:**
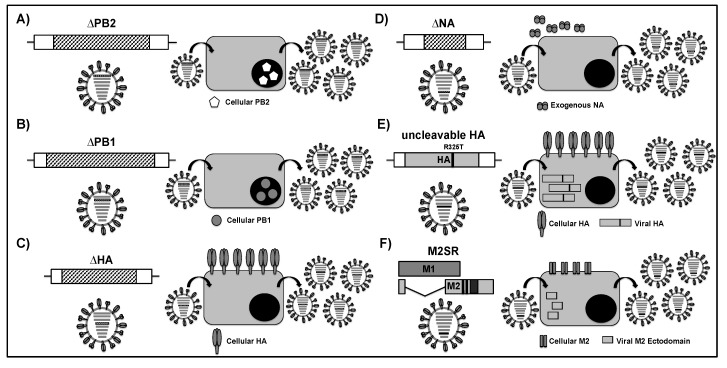
Single-cycle infectious IAV (sciIAV) as LAIV: Schematic representation of sciIAV based on deletions in the PB2 (**A**), PB1 (**B**), HA (**C**), or NA (**D**) viral proteins. SciIAV based on a modified uncleavable HA (**E**) or a non-functional M2, M2SR (**F**) are also indicated. The packaging signals located at the 3’and 5’ ends of each vRNA are indicated with white boxes (**A**–**F**). Lines at the end of each vRNA indicate the 3’and 5’ NCRs (**A**–**F**). Striped boxes represent an internal deletion of the PB2 (**A**), PB1 (**B**), HA (**C**), or NA (**D**) ORF. A black line represents an amino acid substitution (R325T) in the cleavage site of HA that results in an uncleavable HA protein (**E**). A black box represents the deletion of the M2 transmembrane domain (amino acids 25 to 53) that together with the insertion of two stop codons (black lines) downstream of M1 ORF abolish M2 expression (**E**). In the case of ∆PB2 (**A**), ∆PB1 (**B**), ∆HA (**C**), uncleavable HA (**E**), and M2SR (**F**) sciIAVs, efficient viral replication is accomplished by complementation, *in trans*, of the deficient (**A**–**C**) or mutated (**E**–**F**) viral proteins by constitutively expressing PB2 (**A**), PB1 (**B**), HA (**C**,**E**), or M2 (**F**) using stable cell lines. In the case of the ∆NA sciIAV (**D**), exogenous NA is provided in the tissue culture supernatant for the efficient release of infectious viral particles.

In the last few years, different sciIAVs have been described and used in multiple in vitro and in vivo applications, such as the study of segment incorporation and packaging signals [194,195], neutralization assays [196], screening of neutralizing antibodies [106] or antivirals [197], drug resistance [198,199], HA complementation [102], NA functional analysis [200], or the development of vaccines [107,201,202,203,204,205,206,207,208,209,210,211,212,213,214]. The main advantage of using sciIAVs as LAIVs is that host immune response may be elicited without causing the pathogenesis that is associated with viral replication and progeny production [107,205]. In this case, it is important to consider the impact of the missing gene in immunogenicity, because some of the sciIAVs that are deficient in polymerase (PB2, PB1 or PA) segments may have limited levels of viral replication and gene transcription, thus compromising the induction of B or T cell immune responses and protection efficacy.

(**a**) SciIAVs with deletion or substitution of a viral component: The most common strategy to generate a sciIAV is the complete or partial deletion of a viral component. However, it has been shown that the presence of eight viral segments and the vRNA packaging network may be important for optimal virus fitness [195]. To reconstitute the full viral genome, the modified viral gene should contain the packaging signals and NCRs of the native segment, allowing for the insertion of foreign genes (e.g., reporter genes encoding fluorescent or luciferase proteins) [190]. Different sciIAVs have been generated for a diverse range of purposes by deleting PB2 (ΔPB2, Figure 9A) [194,196,201,202,203,204], PB1 (ΔPB1, Figure 9B) [198,199,200], HA (ΔHA, Figure 9C) [102,106,195,205,206,207,209], or NA (ΔNA, Figure 9D) [194,210,211,212,215] viral genes and providing the missing protein *in trans* by generating PB2- (Figure 9A), PB1- (Figure 9B), or HA- (Figure 9C) expressing stable cells lines, or by addition of exogenous NA in the tissue culture supernatant for the efficient release of infectious viral particles in the case of ΔNA (Figure 9D). However, only PB2- [201,202,203,204], HA- [205,206,209], and NA-deficient [210,211,212] sciIAVs have been evaluated as vaccine candidates.

A PR8 sciIAV ΔPB2 (Figure 9A) expressing the green fluorescent protein (GFP) between the NCR and coding 120 nucleotides at both the 5′ and 3′ ends of the PB2 gene was used as vaccine candidate by Victor et al. [201]. This vaccine was shown to be safe in mice and it elicited antibodies against viral proteins as well as GFP, suggesting that the sciIAV ΔPB2 might serve as a LAIV and also as a vaccine vector to deliver antigens from other pathogens in order to develop bivalent vaccines. Although the authors did not find an improvement in protection efficacy of PR8 sciIAVΔPB2 as compared to inactivated WT PR8, they proved that this sciIAV represents a reliable, safe, and efficacious LAIV candidate. Uraki et al. generated a bivalent vaccine by using the PR8 sciIAV ΔPB2 backbone as viral vector and by replacing PB2 with a heterologous IAV HA from either pH1N1 or A/Vietnam/1203/2004 H5N1 [202]. The resultant viruses were immunogenic and protected mice from lethal challenge with PR8, and also the IAV from which the foreign HA originated. Similar results were obtained by other authors who replaced the PB2 vRNA by antigens from different pathogens, such as human parainfluenza virus type 3 (HPIV-3) [203] or respiratory syncytial virus (RSV) [204], suggesting that this platform can be used to develop both monovalent and/or bivalent vaccines against influenza strains or different respiratory pathogens.

In order to study the packaging signals for IAV HA segment, sciIAVs ΔHA (Figure 9C) containing the GFP with HA packaging regions (45 3′-end and 80 5′-end nucleotides) were first developed by Marsh et al. using the backbone of A/WSN/33 H1N1(WSN) or PR8. [195]. SciIAVs ΔHA were efficiently passaged in MDCK cells that constitutively expressed the WSN HA protein, although the virus could not be spread in parental MDCK cells. Using a similar strategy, Martinez-Sobrido et al. [106] demonstrated the use of GFP-expressing sciIAVs ΔHA pseudotyped with different HA proteins for the identification of neutralizing antibodies in monoclonal or polyclonal preparations. Importantly, the newly described fluorescent-based microneutralization assays could be conducted under biosafety level 2 (BSL-2) conditions, even in the identification of neutralizing antibodies against highly pathogenic human or avian IAVs, such as the 1918 Spanish influenza (A/Brevig Mission/18 H1N1) and HPAIV (A/Vietnam/1203/04 H5N1) [106]. In addition, the authors showed that the sciIAV ΔHA have similar morphology when compared with IAV WT [106]. More recently, Baker et al. generated sciIAVs ΔHA using the backbone of IAV pH1N1, PR8, or X31 [205,206,216]. Interestingly, the sciIAV ΔHA pH1N1 stimulated robust immunity in both mice and ferrets, conferring protection against WT viral challenges [205]. Additionally, both sciIAV ΔHA pH1N1 [205] and X31 [206] conferred protection against heterologous infection by eliciting a memory CD8^+^ T-cell immune response. Moreover, Katsura et al. has also used the sciIAV ΔHA system to generate a bivalent vaccine harboring the pneumococcal surface protein A (PspA) from *Streptococcus pneumoniae* with the ability to protect against both IAV and *S. pneumoniae* [209].

The potential of a sciIAV ΔNA (Figure 9D) as an LAIV was firstly evaluated by Shinya et al. [210]. They generated a sciIAV ΔNA in the backbone of WSN by inserting the ORF of GFP between the first 183 (3’ end) and the last 157 (5’ end) nucleotides of the NA vRNA. This virus was attenuated in parental cell cultures, yet it grew efficiently in cell lines expressing reduced levels of sialic acids. The GFP-expressing sciIAV ΔNA was attenuated in mice and it could induce complete protection against a lethal dose of WT WSN. Since the deletion of NA could allow the insertion of a gene encoding a protein antigen from another pathogen, Masic et al. generated a swine influenza virus (SIV) by fusing the H3 HA ectodomain from A/Swine/Texas/4199-2/98 H3N2 to the cytoplasmic tail, transmembrane domain, and stalk region of NA from A/Swine/Saskatchewan/18789/02 H1N1 SIV [211]. This virus was highly attenuated in pigs and it was able to induce H1- and H3-specific immune responses that conferred full protection against WT challenges with H1 and H3 SIVs [211,212]. Moreover, and similar to sciIAV ΔHA, sciIAV ΔNA has been used for the screening of neutralizing antibodies against high pathogenic IAV in BSL-2 facilities [215].

(**b**) SciIAV based on mutations of a viral component: The mutation of certain viral components that results in a loss of functionality, expression, or processing is other strategy that is used to develop sciIAVs, without needing to delete or substitute a complete viral gene [107]. Powell et al. modified the HA sequence that was derived from a PR8 strain (Cambridge) that is particularly virulent for mice by replacing the original ATG start codon with a TAG stop codon to suppress the translation of the signal sequence [207]. In addition, they removed a single nucleotide at the end of the signal sequence to ensure that, if the original ATG was reconstituted from the mutated TAG stop codon, then the sequence would read out of frame. Finally, as a further safety, the HA cleavage site was also inactivated [217]. The resultant recombinant PR8 virus lost all virulence in mice and elicited heterotypic protection that is associated with a robust T-cell immune response [207].

Mutations in the HA cleavage site have also been used as an approach to develop sciIAVs as LAIV candidates [208]. Katsura et al. engineered a reassortment between pH1N1 and PR8 viruses using the HA and NA from pH1N1 and the remaining six segments from PR8. The arginine (R) at the cleavage site of HA1 (position 325), which is critical for HA cleavage by trypsin-like protease, was changed to threonine (T). Since this HA could not be processed into HA1-HA2, which is essential for membrane fusion, the virus was unable to complete its replication cycle. Thus, the MDCK cells that constitutively expressed HA from pH1N1 were used to propagate the virus (Figure 9E). When mice were immunized with this uncleavable HA sciIAV, influenza-specific humoral (IgG and IgA antibodies) and CD8^+^ T-cell responses were more efficiently induced than in mice immunized with the formalin-inactivated IIV. Moreover, mice that were immunized with the uncleavable HA sciIAV survived the lethal WT pH1N1 challenge, while mice immunized with IIV did not.

IAV ion channel M2 protein has an important role in viral assembly and morphogenesis and it is a proven target for anti-influenza drugs [218]. Viruses lacking the cytoplasmic tail domain of M2 have been used as LAIV for different IAVs, such as PR8 H1N1 [219], HPAI H5N1 [220], or pH1N1 [221]. All of the LAIVs provided effective protection in mice against challenge with homologous viral strains. Next, Sarawar et al. developed a second-generation M2-knockout (M2SR) PR8, in which M2 expression was abrogated by deleting the M2 transmembrane domain plus inserting two stop codons downstream of the M1 ORF [214]. The resulting M2SR virus encoded the M2 ectodomain, but it lacked the coding capacity for the M2 transmembrane domain and cytoplasmic tail (Figure 9F). M2SR virus amplification relied on the use of MDCK cells that stably expressed the M2 protein, since M2SR replication was restricted in parental MDCK. However, the virus conserved the ability to elicit systemic and mucosal immune responses that protected mice against homosubtypic (sterilizing) and heterosubtypic (non-sterilizing) challenges [214]. More recently, a similar strategy (Figure 9F) was used to generate a PR8 M2SR containing the HA and NA from a HPAI A/Vietnam/1203/2004 (M2SR H5N1) or a pH1N1 (M2SR H1N1) [213]. The LAIV potential of these viruses was evaluated in both mice and ferrets. In mice, M2SR H5N1 provided sterilizing immunity and both M2SR H5N1 and M2SR H1N1 conferred complete protection with 100% survival after lethal challenge with WT H5N1. Likewise, ferrets that were vaccinated with either M2SR H5N1 or M2SR H1N1 in a prime-boost regime were protected against lethal challenge with WT H5N1. In both animal models, only the M2SR H5N1 provided protection by inducing neutralizing antibodies against H5N1. In contrast, M2SR H1N1 conferred heterologous protection, which might rely on cross-reactive HA2 stalk region and/or NA antibodies. In summary, the authors have shown that the M2SR platform could be used to provide effective homo- and hetero-subtypic protection against IAV in both mice and ferrets [213]. Notably, all of these sciIAVs showed a great potential as LAIVs since mice or ferrets (in the case of M2SR H5N1 and M2SR H1N1) were effectively protected against WT homologous challenges. Currently, a phase I clinical trial is evaluating the safety and immunogenicity of a monovalent M2SR-based LAIV based on 5 gene segments from influenza PR8 and the HA and NA glycoproteins from an A/Brisbane/10/2007-like virus (ClinicalTrials.gov Identifier: NCT03553940).

### 2.6. LAIVs Based on Premature Termination Codon (PTC)-Harboring Viruses

**Figure 10:**
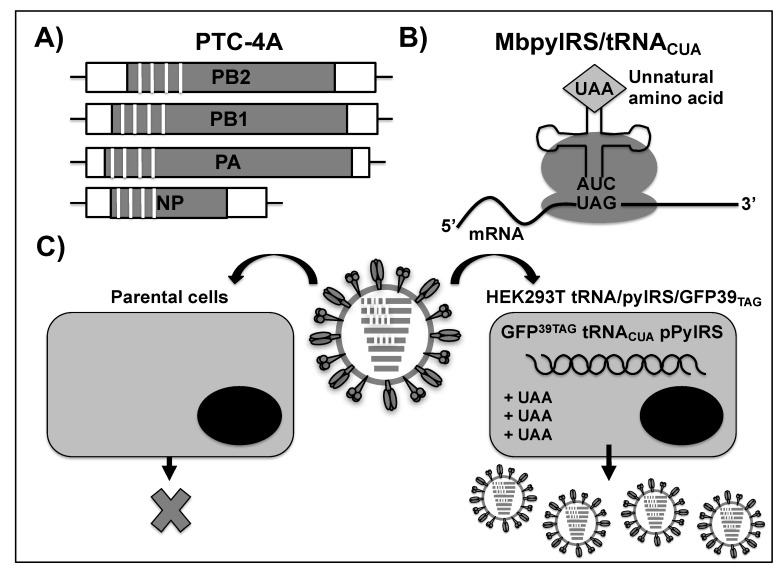
Generation of LAIV based on replication-incompetent viruses: (**A**) Introduction of premature termination codons (PTC) into the viral genome: IAV segments PB2, PB1, PA and NP containing four amber codon substitutions for the generation of replication-incompetent viruses are indicated. Packaging signals of each vRNAs located at 3’and 5’ ends are represented in white boxes. Lines at the end of each vRNA indicate the 3’and 5’ NCR. (**B**) Schematic representation of the orthogonal translation system: Schematic representation of ribosomal incorporation of the orthogonal unnatural amino acid (UAA) and the UAA-tRNA recruitment during the translational elongation event. An UAA is charged onto a tRNA with the required non-sense anticodon by an orthogonal tRNA synthetase. This tRNA then recognizes its corresponding mRNA non-sense codon in the ribosome, leading to the incorporation of the UAA into the protein of interest. (**C**) Establishment of a virion packaging system compatible with the orthogonal translation machinery: Generation of premature termination codon (PTC) viruses are characterized by replication incompetence in conventional cells (left) but efficient replication in cells that contain the cassettes for the expression of orthogonal tRNA (tRNA_CUA_), tRNA synthase (pylRS), and a gene encoding an amber codon–containing GFP (GFP^39TAG^), leading to viral production (right).

Live but replication-incompetent IAV vaccines have recently been generated that are based on the generation of a premature termination codon (PTC) [222]. Normally, the ribosome translates genome sequences into a polypeptide by complementing triplet-codons with matching aminoacylated tRNAs. The triplet UAG (amber codon) does not code for an amino acid and it is usually a translation termination signal or stop codon, resulting in the termination of the synthesis of the nascent polypeptide. However, in certain species, the amber codon corresponds to a tRNA anticodon (tRNA^CUA^), which is able to introduce an unnatural amino acid (UAA) at a stop codon. These UAG-tRNAs have been successfully used in research to introduce UAAs into proteins. In this case, a complementary amber tRNA^CUA^ is aminoacylated by an orthogonal aminoacyl-tRNA synthetase that is designed to only accept UAAs [223]. Longlong Si et al. generated a LAIV using a transgenic human embryonic kidney 293T cell line that contained the orthogonal translation machinery [222]. Transgenic cells contained integrated cassettes for the expression of the *Methanosarcina barkeri* MS pyrrolysyl tRNA synthetase/tRNA^CUA^ pair (pylRS/tRNA_CUA_), the orthogonal UAA N^ε^-2-azidoethyloxycarbonylL-lysine, and a gene encoding an amber codon-containing GFP (GFP^39TAG^) (Figure 10). GFP expression in the presence of UAAs was used to select genetically stable cells lines after 200 passages. The ability of the resultant transgenic cells (HEK293T-tRNA/pylRS/ GFP^39TAG^) to propagate a WT WSN virus was tested in parallel with parental HEK293T cells. Subsequently, different PTC viruses were generated, harboring amber codons in different viral segments, although a PTC virus harboring four stop codons in PA, PB2, PB1, and NP (PTC-4;) segments was used, since it maintained the intact surface antigens HA and NA (Figure 10A). All of the PTC viruses were generated in transgenic cells expressing pylRS/tRNA_CUA_, which were able to introduce UAA at each stop codon position (Figure 10B). The generation and stepwise production of PTC viruses were only possible in the transgenic cells in the presence of UAAs and not in the parental HEK293T cells (Figure 10C), suggesting a high level of safety for this technology as LAIV. The safety of PTC-4 virus was evaluated by intranasally infecting mice, ferrets, and guinea pigs. The results that were obtained from the three animal models indicated that PTC-4 virus was attenuated and reversion did not occur in vivo. Moreover, the PTC-4 virus induced a robust mucosal, humoral, and cell-mediated immunity that conferred protection against homologous WT challenge in the three animal models [222]. In addition, PTC-4 protected mice from a challenge with heterologous IAVs. Together, these results confirm that replication-incompetent viruses based on PTC technology can potentially be implemented as LAIV to confer protection against both homosubtypic and heterosubtypic IAV strains. Due to the simplicity of the technique, it can be adapted to almost any virus with a genome that can be manipulated and packaged in a cell line.

## 3. Conclusions and Future Directions

Vaccination remains the primary and most cost-effective strategy to prevent and control influenza infections in human and animal populations. The continued evaluation of the safety, immunogenicity, and effectiveness of seasonal influenza vaccines is needed to ensure low rates of influenza infection among children and adults. However, efforts should be made to improve the vaccine development process to allow for a shorter timeline from the initial identification of the WHO candidate to the final distribution of the vaccine. In general, LAIVs are more immunogenic and they provide better protection than their IIV counterparts. However, in past influenza seasons, a suboptimal protection was observed mainly against the pH1N1, leading the ACIP to revoke the LAIV recommendation in the following seasons. Although the LAIV was approved for use during the 2018–2019 influenza season, the reduced effectiveness of the quadrivalent LAIV remains unknown. This highlights the urgent and pressing medical need of new approaches to generate more effective LAIVs that are capable of preventing or ameliorating the effects of seasonal and/or pandemic influenza by providing broader and more long-lasting immune protection than that elicited by current vaccines. This need is reflected in the current priorities of the National Institute of Allergy and Infectious Diseases (NIAID) and the Centers of Excellence of Influenza Research and Surveillance (CEIRS),, as exemplified by the strategic plan that was recently proposed for developing a universal influenza vaccine [224]. This universal vaccine should be at least 75% effective against symptomatic infection, protect against group 1 and 2 IAVs during at least one year and preferably multiple seasons, and be suitable for all age groups [224]. In terms of protection, the ability of LAIV to replicate in the upper respiratory tract is an advantage for inducing the broader and long-lasting immune response that is expected from a universal vaccine [225].

The implementation of reverse genetics approaches has impacted the development of LAIVs by reducing the timeline that is needed for vaccine manufacturing, which is especially relevant in the generation of LAIVs against pandemic IAV strains. Ideally, future vaccines should increase the breadth of immune responses to protect against antigenically different IAVs within the same or different subtypes and it should be able to be manufactured in cells lines to further reduce production time. Here, we have provided an update on the progress that has been made in the influenza vaccination field in the last five years to improve or overcome current vaccine production and effectiveness drawbacks. The potential of implementing these approaches for the development of a universal vaccine that would elicit immune responses with broad specificity and providing long-acting protection, which is one of the current and major priorities of the NIAID, and the CEIRS, must be highlighted.
